# Impact of Partially Automated Driving Functions on Forensic Accident Reconstruction: A Simulator Study on Driver Reaction Behavior in the Event of a Malfunctioning System Behavior

**DOI:** 10.3390/s23249785

**Published:** 2023-12-12

**Authors:** Daniel Paula, Maximilian Bauder, Claus Pfeilschifter, Franziska Petermeier, Tibor Kubjatko, Klaus Böhm, Andreas Riener, Hans-Georg Schweiger

**Affiliations:** 1 CARISSMA Institute of Electric, Connected, and Secure Mobility, Technische Hochschule Ingolstadt, Esplanade 10, 85049 Ingolstadt, Germany; maximilian.bauder@carissma.eu (M.B.); hans-georg.schweiger@thi.de (H.-G.S.); 2 CARISSMA Institute of Automated Driving, Technische Hochschule Ingolstadt, Esplanade 10, 85049 Ingolstadt, Germanyandreas.riener@thi.de (A.R.); 3 Institute of Forensic Research and Education, University of Zilina, 010 26 Zilina, Slovakia; 4 Department of Mechanical, Automotive, and Aeronautical Engineering, Munich University of Applied Sciences, Dachauerstraße 98b, 80335 Munich, Germany; klaus.boehm@hm.edu

**Keywords:** accident analysis, partially automated driving functions, driver reaction behavior

## Abstract

Partially automated driving functions (SAE Level 2) can control a vehicle’s longitudinal and lateral movements. However, taking over the driving task involves automation risks that the driver must manage. In severe accidents, the driver’s ability to avoid a collision must be assessed, considering their expected reaction behavior. The primary goal of this study is to generate essential data on driver reaction behavior in case of malfunctions in partially automated driving functions for use in legal affairs. A simulator study with two scenarios involving 32 subjects was conducted for this purpose. The first scenario investigated driver reactions to system limitations during cornering. The results show that none of the subjects could avoid leaving their lane and moving into the oncoming lane and, therefore, could not control the situation safely. Due to partial automation, we could also identify a new part of the reaction time, the hands-on time, which leads to increased steering reaction times of 1.18 to 1.74 s. The second scenario examined driver responses to phantom braking caused by AEBS. We found that 25 of the 32 subjects could not override the phantom braking by pressing the accelerator pedal, although 16 subjects were informed about the system analog to the actual vehicle manuals. Overall, the study suggests that the current legal perspective on vehicle control and the expected driver reaction behavior for accident avoidance should be reconsidered.

## 1. Introduction

Installing advanced driver assistance systems (ADASs) in vehicles is intended to increase road safety and driving comfort [1,2]. There are vehicles on the market today with partially automated driving functions (SAE Level 2) on board [3]. These systems can take over both longitudinal and lateral control of the vehicle for a limited period of time. Taking over the driving task is associated with automation risks resulting from limitations or malfunctions the driver must control [4]. For example, studies on the performance of partially automated driving functions have revealed that vehicles can deviate from their own lane into the opposite lane when cornering [5]. In addition, recent fatal traffic accidents, for which Tesla must justify itself in US courts [6], demonstrate that ADASs are also limited in their ability to recognize objects. For example, autonomous emergency braking systems (AEBSs) have incorrectly initiated braking actions (so-called phantom braking) when the vehicle was moving in partially automated driving mode, which led to serious rear-end collisions [7].

To clarify such accidents, the investigating authorities often consult experts. Their tasks include determining the accident’s cause and the parties’ driving behavior. Furthermore, the avoidability of the collision for the parties involved must be considered, in which the expected reaction behavior of the driver from a legal point of view is a decisive factor for the results of the simulation calculations [8]. From a legal point of view, the driver is responsible for vehicle control at all times, even if an ADAS is activated and influences dynamic driving behavior (possibly incorrectly) [9]. As a result, possible ADAS–driver interactions are currently not considered in forensic accident reconstruction, and the standardized reaction behavior of the driver, which was determined during manual driving and consequently without taking partially automated driving functions into account, is used. Furthermore, the results of a survey conducted with 173 internationally active accident reconstruction experts [10] showed also a lack of reliable basic data on the driver’s performance in the event of erroneous behavior of an activated partially automated driving function, since this has only been investigated in a few studies with differing results [11,12,13,14,15].

On the other hand, due to Revision 1 of UN Regulation No. 160 [16], which came into force in October 2022, ADAS activities that took place up to 5 s before the collision will be stored in newly produced vehicles in the future. With the availability of this data on ADAS activities, it can be assumed that more and more parties involved in accidents will demand that ADAS–driver interactions have to be examined in forensic investigations, especially if ADAS misbehavior is a possible cause of the accident. Consequently, the necessity and, thus, the central objective of the present work is to generate fundamental data on driver reaction behavior in the event of malfunctioning behavior of partially automated driving functions. In particular, conclusions are generated for forensic accident analysis whether, in the case of an activated partially automated driving function during an accident, a change in the driver’s reaction behavior compared to manual driving must be considered. Therefore, this work investigates the reaction behavior of drivers in the case of a system limitation of an activated partially automated driving function during cornering. Secondly, findings are generated on the behavior of drivers in the event of phantom braking initiated by the AEBS and whether they can correctly override the system’s misbehavior while a partially automated driving function is activated at the same time.

### 1.1. Driver Reaction Behavior

**Figure 1:**
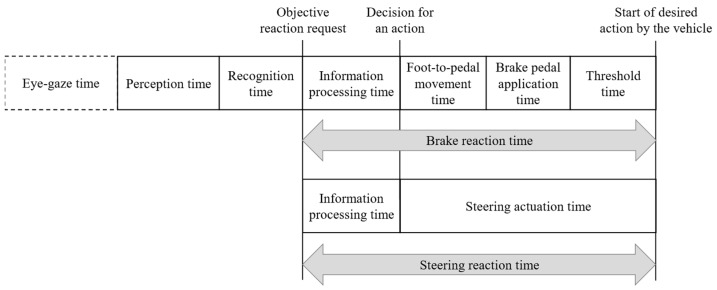
Sequences of a brake and steering reaction time (own illustration according to [8,18]).

For assessing liability in the context of a judicial appraisal of a traffic accident, the expert must consider the conditions in which the collision could have been avoided by the parties involved. From a legal perspective, the expected reaction behavior of the driver must be applied. According to Figure 1, a driver’s reaction is the response to a previous environmental change, representing a hazardous situation. Consequently, it initiates the start of a dangerous driving situation. The driver must first perceive the environmental change or hazard (perception time) and recognize it (recognition time). The prerequisite is that the object is in the driver’s foveal field of vision without a required movement of the driver’s gaze.

In forensic accident analysis, the objective reaction request point is when the driver must have recognized the danger, which leads to a reaction. The information processing time starts with the objective reaction request and the associated recognition of the necessity to initiate a defensive action. Within this time, the driver decides about the type of action (e.g., braking or steering). This duration can vary greatly and depends on the situation’s urgency and the driver’s expectations [17]. After combining the results of several studies, values for the information processing time of 0.45 s in the arithmetic mean, 0.22 s for the 2% quantile and 0.58 s for the 98% quantile were found [18]. The decision-making time for the performed type of action is followed by the foot-to-pedal movement time in the case of a braking reaction. In the case of a braking reaction, the foot-to-pedal movement time starts with the decision for an action. According to the state of the art, this time is defined as the time from when the accelerator is released until the brake pedal is touched. The time range is usually between 0.15 s and 0.3 s [8]. The foot-to-pedal movement time is again followed by the phases of brake pedal application time and threshold duration [8,19]. According to Figure 1, a pure steering reaction time does not include all these time sequences but does include steering actuation time. This includes the time required for the driver to realize the required steering wheel angle and the time required for the mechanical transfer of the steering wheel angle executed by the driver to the individual wheels. The upper limit of the steering wheel angular velocity is approximately 400°/s [8]. At the end of the braking and steering reaction time, the vehicle starts to perform the desired action, and the associated changes in driving dynamics in the longitudinal or lateral direction begin.

The explanations demonstrate that the driver’s reaction time depends on numerous factors. Consequently, many different results are available from test series and simulator studies. In the report “Behavior-related characteristics of time-critical driving maneuvers” published by the German Federal Highway Research Institute [20], 160 international sources on reaction time tests and studies were analyzed, evaluated, and compared. In summary, the authors found that the driver’s expectation of being forced to react plays a decisive role. Accordingly, the braking reaction time of a driver is approximately 0.7 s in the case of a high expectation and 1.0–1.5 s in the case of surprising events. The report also stated that steering reaction times are approximately 0.1 s to 0.2 s shorter than braking reaction times [20]. The urgency of the reaction influences the driver’s braking and steering reaction times, typically in the order of 0.2 s to 0.3 s [20].

In summary, for an urgent and surprising event for the driver, which is usually the case in a severe imminent accident, a braking reaction time range of 0.8 s to 1.3 s and a steering reaction time range of 0.6 s to 1.2 s can be expected. The listed fundamental data on driver reaction times were determined with manual steering control by the driver and thus without considering partially automated driving functions.

### 1.2. Related Work

Studies have already been carried out on driver performance when it is necessary to take over driving control because of a partially automated driving function malfunction. M. S. Young et al. found a 1.0 s to 1.5 s longer braking reaction time of the driver compared to manual driving when a partially automated driving function (adaptive cruise control (ACC) system + lane centering assistance (LCA) system) is activated and does not react to a braking vehicle in front [21]. The authors in [22] found that drivers who know the possible system limits and have experience with such a system can also have a braking reaction time similar to that of manual driving. Shen et al. investigated drivers reacting to the misbehavior of a partially automated driving function by leaving their lane and moving into the oncoming lane in a straight section of the road due to a sudden strong crosswind [13]. The results showed a significantly longer steering reaction time (M = 1.27 s, SD = 0.61 s) for subjects who drove in partially automated mode than those who steered the vehicle manually (M = 0.69 s, SD = 0.30 s). In the case of additional non-driving activities being performed by the driver while the Level 2 system was activated, the driver’s reaction time was increased by up to 0.3 s, comparable to the findings in [20]. In a further study, Shen and Neyens investigated the reaction of test subjects in the event of a sudden failure of the LCA system during straight-ahead driving without oncoming traffic, cornering without oncoming traffic, and straight-ahead driving with oncoming traffic [12]. Again, longer steering reaction times were found compared to manual driving.

These findings confirm the results of several studies [23,24,25,26]. In this study, the authors found that a driver’s attention to the traffic situation decreases significantly when a partially automated driving function is activated and confidence in the system’s performance has been built up. This can be attributed to the fact that the driver is actively released from the control loop when the system is used and is, therefore, tempted to become inattentive. The main reason for this is that following UNECE Regulation 79 [27], the driver has the technical option of taking both hands off the steering wheel for 15 s and glancing away from the road. This “out-of-the-loop” problem has been documented in several other studies that found impaired driver performance in regaining driving control with an unexpected malfunction of a partially automated driving function [28,29,30]. The fact that the driver must return the hands to the steering wheel when a critical driving situation occurs can also explain the longer steering reaction times compared to manual driving, as evidence was found of an additional “hands-on time” of around 0.3 s [31]. The IIHS long-term study also revealed that drivers increasingly use the technical option of taking their hands off the steering wheel for up to 15 s the more familiar they become with the partially automated driving function [23]. Dikmen M. found from a survey conducted among Tesla drivers that drivers build up increasing trust in the systems over time and, as a result, overestimate their performance [32]. Furthermore, in evaluating several studies, N. Strand reported that human drivers are generally quick to trust a functioning vehicle system and are generally unsuited to monitoring (partially) automated systems, especially if they cause a dangerous driving situation [15]. This finding also corresponds with the results of a survey conducted by the AAA Foundation for Traffic Safety, in which 90% of the participants stated that they owned a vehicle equipped with a partially automated driving function and used it regularly but did not know or understand the system limits described in the manual and had not informed themselves about the system functionalities in any other way [33].

The related work shows that several studies have already been conducted on partially automated driving functions, in which a change in the driver’s reaction behavior was found compared to manual driving. However, in all studies, individual factors relevant to forensic accident reconstruction were not sufficiently considered, so the fundamental data obtained cannot be used beyond doubt in legal disputes. No system behavior that can verifiably occur in real vehicles was modeled in the test scenarios of the simulator studies. Consequently, before the present study, a VW Travel Assist [34] and Tesla Autopilot [35] were used to investigate how a vehicle can move from its lane into the oncoming lane while driving in partially automated mode. The results published in [5] revealed that this can occur in tight curves due to the limited lateral acceleration the system is allowed to apply, to a maximum of 3 m/s^2^ [27]. In addition to the driving dynamics parameters, the system’s messages to the driver were also recorded during the tests. It was found that the warning message “Take over steering immediately“ only appears after the center line has been crossed and not, as in [11], before the center line is crossed. It can be assumed this leads to a significant change in the driver’s reaction behavior. In [11], the period from 15 s to the recurring request to touch the steering wheel also varied, which is inconsistent with actual system behavior [36]. At the same time, an increasing “out-of-the-loop” effect can decrease the driver’s attention to the driving situation. In [15,37], the test subjects were distracted by a secondary task. As expected, this led to longer reaction times being measured. In the study by D. Damböck, in which a “hands-on time” of 0.3 s was measured, the test subjects had to perform a secondary task when the vehicle was moving on a country road in partially automated mode [31]. In addition, the study by D. Damböck focused on investigating the reaction behavior of drivers to an animal crossing a straight road. The time to collision with the animal was six seconds, and there was no oncoming traffic, which might have reduced the urgency of touching the steering wheel. Measured values in which test subjects had to conduct a secondary activity cannot generally be used in forensic accident reconstruction. This is because the determined reaction times of ideal drivers who were neither inattentive nor distracted from the actual driving task must be applied by law. A delayed reaction results from avoidability analysis, from which the driver’s inattention and, thus, an increased proportion of fault can be derived. Also, no oncoming traffic was modeled in the studies cited regarding the system’s misbehavior when cornering. This can influence the urgency of the driver’s reaction and increase reaction time by 0.2 s to 0.3 s. 

Furthermore, the literature has showed that a driver’s reaction behavior during system-initiated phantom braking is entirely unexplored. Of particular importance is the fact that the braking maneuver, when it is performed incorrectly and without any apparent reason for the driver, must be overridden by the driver by pressing the accelerator pedal [38]. This is an entirely different reaction pattern compared to a classic maneuver (braking/steering) which previously had to be performed by the driver in critical situations. Therefore, this work represents an absolute novelty in the literature regarding accident analysis, partially automated driving functions, and corresponding human factors.

### 1.3. Research Questions

The previous explanations demonstrate that the misbehavior of an activated, partially automated driving function can result in a dangerous situation for road users. From a legal perspective, it is assumed that the driver must always be able to control the vehicle. This also applies in the event of a system malfunction. Therefore, the driver must perform permanent system monitoring. This implies that the occurrence of a system limitation must be immediately recognized by the driver and lead to a reaction. At the same time, however, the studies found a change in driver behavior when using a partially automated driving function compared to manual driving. As a result, it is questionable whether the driver can even fulfill his responsibility to immediately take control of the vehicle in case of a system malfunction. 

The literature has shown that there is a lack of fundamental data on the reaction behavior of drivers when a system limitation of an activated partially automated driving function occurs. This is particularly the case during cornering and phantom braking. Furthermore, according to the findings from [5], it cannot be assumed that the system will alert the driver sufficiently far in advance about the upcoming system limitation while considering an appropriate takeover time. Based on this, the following research questions were specified:

RQ1: When do drivers recognize the misbehavior of the activated partially automated driving function?
RQ2: What time do drivers need from the misbehavior recognition to the start of the overruling action?
RQ3: Is it possible for the driver to avert a critical driving situation with the initial overruling action?

## 2. Materials and Methods

Since the driver’s reaction behavior in a hazardous situation was investigated in both situations, it was only feasible to conduct the tests in a driving simulator. Although the test scenarios were modeled as realistically as possible with great effort, a simulator cannot fully reproduce reality in all its variations [39]. Nevertheless, studies have shown that the driver’s behavior and decisions can be transferred very well from the results of a simulator study to real situations [40].

### 2.1. Driving Simulator

The study was conducted in a highly realistic driving simulator at Technical University Ingolstadt of Applied Sciences. The simulator consists of a shortened VW Golf 5 mounted on a hydraulic platform [41]. This allows for realistic driving scenarios with all six degrees of freedom to be dynamically simulated with the aid of four projectors. The technical data of the simulator and details on the hardware and software setup can be found in [42]. The suitability of the driving simulator for conducting scientific studies has often been demonstrated in recent years [43,44,45].

The driving simulator features an integrated communication system, pedals for the accelerator and brakes, and a highly sensitive steering system with force feedback. Inside the VW Golf 5, a head-up display (HUD) was installed with a specially coated acrylic panel on a tablet computer. The speedometer consists of a vehicle display powered by a Raspberry Pi 3. IPG CarMaker 10.2.2 was used to design the roads and test scenarios (driving maneuvers, etc.) and as simulation software [46]. CarMaker also recorded the measurement data relevant to the investigation of the research questions. These were the accelerator pedal position, brake pedal position, steering wheel angle, distance traveled, position on the road, driving time, vehicle speed, and vehicle acceleration in longitudinal and lateral directions.

**Figure 2:**
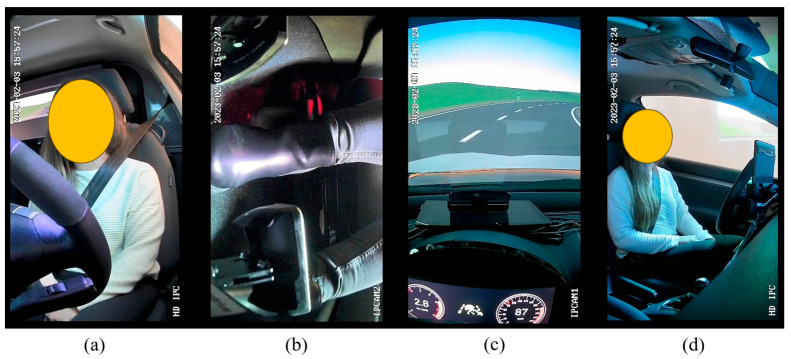
Camera views during a study run. From left to right: view of the driver from the front (**a**); view of the pedals (**b**); view of the driver on the HUD, the speedometer display, and the windshield (**c**); side view of the driver (**d**).

As shown in Figure 2, four cameras mounted in the vehicle interior were used for the study to record the pedals, the steering wheel, the test subjects, and the information on the speedometer display and HUD, as well as the position of the vehicle on the road using the OBS Studio software 29.1. 

### 2.2. Test Scenarios

The simulator study aimed to investigate driver reaction behavior in the event of ADAS misbehavior. For this purpose, a scenario was defined in which the vehicle with an activated partially automated driving function deviated from its lane into the oncoming lane while cornering on a country road. In the second scenario, a partially automated driving function was also activated, and phantom braking initiated by the AEBS was triggered.

#### 2.2.1. Test Scenario 1: Deviating from Own Lane into the Oncoming Lane while Cornering

To realistically reproduce the actual system behavior that occurs in public road traffic in the driving simulator, preliminary driving tests were conducted on a curving country road, which the driver had not travelled before, with a VW Travel Assist and Tesla Autopilot with the latest software version [5]. The driving trajectories of the vehicles and system activities (e.g., warning messages to the driver) recorded via measurement and video systems were used for programming the vehicle and system behavior in the simulation environment. Map data from the driving tests were used to model the test route. Vehicles approached in the opposite lane at different intervals to make the driving experience as realistic as possible. The road was not very busy, but the test subjects were constantly reminded that there were other road users on the route, which should positively affect the test subjects’ consistently high level of attention.

Forensic accident analysis requires fundamental data on reaction behavior in which the driver experiences an unexpected event similar to what would be expected in an accident. Accordingly, the test scenario “deviating from one lane into the oncoming lane while cornering” was modeled; the test subjects traveled 8718 m before the dangerous driving situation occurred. Over this period, straight sections, as well as curves with different radii, were driven through. The partially automated driving function performed its intended function and consequently showed no misbehavior, which was intended to strengthen the confidence of the test subjects in the system functionality. Furthermore, this design was intended to achieve the natural behavior of the test subjects until they were in danger of leaving their own lane. In long-term studies, it was observed that drivers increasingly used the technical option of taking their hands off the steering wheel for up to 15 s after building up confidence in the system [23].

**Figure 3:**
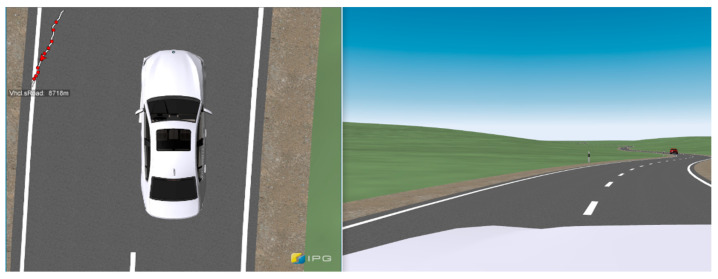
Top view on the left and driver view on the right at the situation when leaving the own lane into the oncoming lane with oncoming traffic.

After traveling the distance to the actual test scenario, the vehicle reached a curve with a radius of 190 m with the partially automated driving function activated, analogous to the test drives carried out. The maximum lateral acceleration the system could achieve was limited to 3 m/s^2^ per UNECE Regulation R79 [27]. The combination of driving speed, curve radius, and lateral acceleration resulted in a departure from the vehicle’s lane. The situation when leaving the driver’s own lane into the oncoming lane with oncoming traffic is shown in Figure 3. This was identical to the real tests and occurred in the simulation due to reaching the curve limit speed limited by the specified maximum lateral acceleration in the curve apex. The prerequisite was that the driver did not override the system in advance and consequently took over vehicle control. The system was not deactivated, and no warning message was sent to the driver when crossing the center line, comparable to the system behavior from the tests [5]. The warning appeared on the arithmetic mean 0.35 s (σ = 0.04 s) after leaving the own lane into the oncoming lane. This was implemented in the simulation software so that a contact between the vehicle and the oncoming lane has activated a timer, whose expiry (M = 0.35 s) causes the warning message to appear in the HUD.

To create a situation comparable to a real accident, the test subjects were forced by a model of an oncoming vehicle to avoid leaving their lane or return as quickly as possible. When crossing the center line, the oncoming vehicle was located 140 m (σ = 24.3 m) from the ego vehicle, and the time to collision (TTC) was 2.75 s (σ = 0.13 s). Furthermore, the activated partially automated driving function did not perform any braking or active steering action back to the driver’s own lane, comparable to the real test results. Instead, the driving trajectory with the corresponding driving dynamics parameters was kept constant, which would lead to a collision with oncoming traffic if the driver did not overridden the system.

The design described aimed to generate a “best-case” scenario. Consequently, the test subjects were not distracted and faced high urgency. This allowed for the determination of reaction times and drivers’ behavior under both idealized and realistic conditions. As a result, this study design determines the driver’s reaction behavior to use it in the future to reconstruct oncoming traffic or roadside collisions. If necessary, these results can be supplemented with additional factors known from other studies, such as distraction, secondary activities, etc., to determine the result of the avoidability analysis and determine whether such factors could have had an additional effect on driver behavior.

#### 2.2.2. Test Scenario 2: Phantom Braking

**Figure 4:**
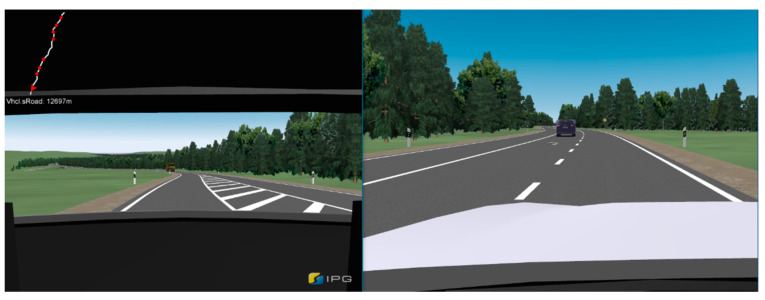
View from rear-view mirror with truck approaching on the left and view through windscreen shortly before phantom braking on the right.

The second test scenario uses a route profile identical to the first test scenario. When approaching the test scenario, the vehicle drives in partially automated driving mode as long as the driver does not override the system. Another vehicle drives in front of the vehicle at a sufficient distance so that the ACC system does not have to reduce the speed of the ego vehicle. The vehicle in front changes from the right-hand lane to a left-hand turning lane and decelerates its speed for the turning maneuver. The ego vehicle continues driving straight ahead in the right-hand lane. Before passing the turning vehicle, according to right-hand illustration of Figure 4, the driving system incorrectly recognizes it as a stationary object in its driving trajectory, triggering a false-positive activation of the AEBS (phantom braking).

Like the first scenario, the goal was to create a “best-case” scenario for investigating the reaction behavior of the test subjects. To increase the urgency for the driver to avoid an imminent rear-end collision when braking to a standstill, a truck following the ego vehicle was modeled, which was visible to the subject via the rear-view mirror (see left-hand illustration of Figure 4). Before reaching the test scenario, the test subjects passed this truck driving on an acceleration lane next to the ego vehicles lane. This way, the test subjects’ awareness of the truck following the ego vehicle should be increased.

### 2.3. System Description

The partially automated driving function (SAE Level 2) could perform longitudinal and lateral vehicle control over the entire test route within the system limits. The maximum permitted speed on German country roads is 100 km/h. Consequently, the target speed of the partially automated driving function was 100 km/h; the speed was regulated automatically to ensure safe cornering.

**Figure 5:**
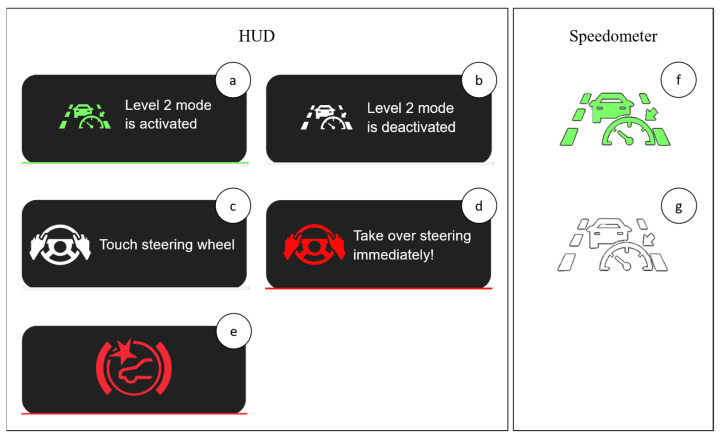
System information in HUD: (**a**) Partially automated driving system activated; (**b**) Partially automated driving system deactivated; (**c**) Touch steering wheel; (**d**) Take over steering immediately!; (**e**) Collision warning. System information in speedometer: (**f**) Partially automated driving system active; (**g**) Partially automated driving system inactive. Messages are according to Volkswagen’s design [47].

At the start of the simulation, the vehicle was stationary in its lane, and the Level 2 system was deactivated, which was visually indicated to the test subjects both in the HUD and the speedometer (Figure 5b,g). Subsequently, the display in the HUD changed, and the message “Touch steering wheel” appeared (Figure 5c). At the same time, an acoustic signal was emitted, like the acoustic signal emitted when Travel Assist is activated in conventional VW Group vehicles. Afterward, the vehicle began to accelerate independently and align itself in the middle of the lane. In parallel, the “Autopilot activated” message in the HUD disappeared, and the permanent message “Autopilot active” was shown in the speedometer (Figure 5f).

The test person could override the partially automated driving function at any time by turning the steering wheel (steering wheel angle ≥ 5°) or pressing the brake or accelerator pedal. In this case, the message “Autopilot deactivated” immediately appeared in the HUD (Figure 5b), the speedometer indication changed to “Autopilot inactive” (Figure 5g), and an acoustic signal was emitted. While the test person controlled the vehicle, the partially automated driving function remained in standby mode, similar to the VW Travel Assist design. As soon as the system detected that the vehicle was back in the center of the lane and the driver had finished acting (steering wheel angle < 5° and no pedal pressing), the system was activated automatically. As a result, the message “Autopilot activated” appeared in the HUD (Figure 5a), and the acoustic signal was emitted simultaneously. Afterward, the message in the HUD disappeared, and the permanent message “Autopilot active” reappeared in the speedometer (Figure 5f).

The test subjects were instructed to use the system. However, they were free to decide where they wanted their hands to be placed. A capacitive sensor activated a timer if the test subject removed the hands from the steering wheel. By UNECE regulation R79 [23], after a hands-off time of 15 s, a request to confirm presence appeared in the HUD in the form of the message “Touch steering wheel” (Figure 5c). If the test person followed this request, the capacitive sensor detected a “hands-on“, the HUD message changed back to “Autopilot activated,” and the timer was reset. This loop was repeated for the entire test run. If the test person did not follow the request to confirm the presence, the message “Take over steering immediately!” (Figure 5d) appeared after 30 s by UNECE regulation R79 [23], and an acoustic warning tone similar to the VW Travel Assist warning tone rang. If the test person continued not touching the steering wheel within the next 15 s, the partially automated driving function was deactivated with the corresponding visual messages in the HUD and speedometer. The display “Take over steering immediately!” (Figure 5d) also appeared in the HUD 0.35 s after crossing the center line during scenario 1. Furthermore, an acoustic warning signal was also emitted when the visual warning message appeared. The visual warning message thus appeared 0.65 s earlier than determined in the real driving tests [4]. This was intended to additionally increase the urgency for the test subjects to act at an early stage and, at the same time, slightly defuse the scenario.

In the second scenario, the driver experienced a braking action initiated incorrectly by the autonomous emergency braking system (AEBS) when the vehicle moved into partially automated driving mode. When the phantom braking was triggered, a visual collision warning message (Figure 5e) was displayed in the HUD, and an acoustic warning tone was emitted. According to UN Regulation No. 152, “Uniform provisions concerning the approval of motor vehicles regarding the Advanced Emergency Braking System (AEBS) for M1 and N1 vehicles” [48], an AEBS must perform a braking deceleration of at least 5 m/s^2^, which was implemented accordingly in the system design. According to the previous explanations, the braking maneuver is performed erroneously and for no apparent reason for the driver and can only be overridden by pressing the accelerator pedal.

### 2.4. Participants

A questionnaire was created for the recruitment of test subjects. This questionnaire interviewed potential test subjects about their experience with advanced driver assistance systems (ADAS). Furthermore, information on annual mileage, gender, and age was requested. Based on the information on experience with ADAS and annual mileage, the subjects were assigned to one of two groups: “experienced with ADAS” and “inexperienced with ADAS”. A total of 32 subjects were invited to participate in the study, 16 of whom belonged to the “experienced with ADAS” group and 16 to the “inexperienced with ADAS” group. For a gender-independent evaluation of the research results, half of one group consisted of women (8) and the other half of men (8). As people aged 55 years and older show longer reaction times [49,50], subjects with an average age of 29.3 years (standard deviation σ = 4.98 years) in the range of 24 years to 46 years were selected for the study, which means that possible age-related effects on the study results can be excluded. All study participants had a valid class B driving license and an average driving experience of 11.5 years (σ = 4.99 years). Almost half of the test subjects reported an annual mileage of over 10,000 km. The study lasted approximately 60 min for each subject. Since another study was conducted in parallel to this study with the same subjects [42], the test blocks were conducted alternately with randomly assigned subjects to avoid a systematic bias of the results due to the study design. The University of Žilina ethics committee reviewed and approved the study before it was conducted.

### 2.5. Instructions and Procedures

Before the study, all test subjects received a service agreement and a consent and data protection sheet, which they had to read through and sign as a condition of participation. In a survey conducted by the American Automobile Association (AAA) Foundation for Traffic Safety, it was found that 90% of drivers who regularly use ADAS and thus show a high level of experience have never read the functionalities and associated limitations of ADAS described in the vehicle manual and have not informed themselves about them in any other way [33]. To ensure that the group of experienced study participants were aware of the system limitations and possibilities for overriding faulty ADAS behavior, they were also given an information sheet describing the functionality of the partially automated driving function and AEBS as well as their limits and possibilities for overriding, based on the VW [38] and Tesla [51] manuals.

A safety briefing on the laboratory and driving simulator followed this. After this, the subject took a seat in the driver’s seat of the driving simulator to adjust its position and the rear-view mirror. The subjects were also briefed on the functionality of the driving simulator and informed that the study could be interrupted at any time. The test subjects then conducted an introductory lap to familiarize themselves with the system functionality and the behavior of the driving simulator. Any questions during this time could be asked directly to the study leaders in the control center via the speakerphone in the driving simulator.

The first test scenario was started after the subject signaled their readiness to stop the introductory round. Due to the randomization, 16 subjects started with test scenario 1 and 16 subjects with test scenario 2, of which eight subjects belonged to the experienced ADAS group and eight subjects belonged to the inexperienced ADAS group. After completing test scenario 1 or test scenario 2, the simulation was continued without interruption so that the subjects reached test scenario 2 or test scenario 1. After the subjects had completed the two test scenarios, they were asked to get out of the vehicle and complete a questionnaire.

### 2.6. Questionnaire

The first question of the questionnaire identified whether the subjects had recognized a critical situation, which could be answered with the fields “yes” and “no”. If the answer field “yes” was selected, the subjects could use a free text field to describe which critical situations they had experienced. 

Subsequently, the subjects were asked how they recognized that they had to intervene in the first situation they experienced (test scenario 1). The response fields (1) Vehicle close to the center lane; (2) Crossing the center lane; (3) High speed; and (4) System warning were available for selection, and several of these could be selected. The subjects also had the opportunity to provide further answers via a free text field. The subjects could subsequently use the “yes” and “no” response fields to indicate whether the situation was controllable. Afterwards, the subjective criticality was queried using a typical five-level Likert item [52], in which the subjects could choose between the response fields (1) Strongly Uncritical; (2) Uncritical; (3) Neither Uncritical Nor Critical; (4) Critical; (5) Strongly Critical. At the same time, the reason for the selection could be stated in a free text field. The last question on test scenario 1 verified the subject’s experience as stated in the recruitment questionnaire by asking whether such a situation had already been experienced in reality when using an ADAS, which could be answered with “yes” or “no”.

For test scenario 2 (phantom braking), the subjects were asked whether they recognized the need to intervene because of the visual warning, the acoustic warning, or braking. After this, the subjects were asked about the controllability and criticality of scenario 2, similar to scenario 1. Thereupon, the subjects were asked whether they had been aware of the presence of a truck in the back, whether they had immediately known how to override a faulty brake intervention, and, if so, how they had known. Finally, the subjects were asked in general whether they would switch off the system permanently if such misbehavior became more frequent and whether they thought such situations should be trained for in driving school.

### 2.7. Dependent Variables

To answer RQ1, whether the subjects had activated the partially automated driving function during cornering was investigated. For the remaining number of subjects who had activated the partially automated driving function, it was examined whether they recognized the system malfunction at the moment of crossing the center line (see Figure 3) or when the vehicle had already left its lane into the oncoming lane, and the warning message appeared. The objective reaction request point was verified with the information provided by the subjects in the questionnaire on the subjective reaction request point.

**Figure 6:**
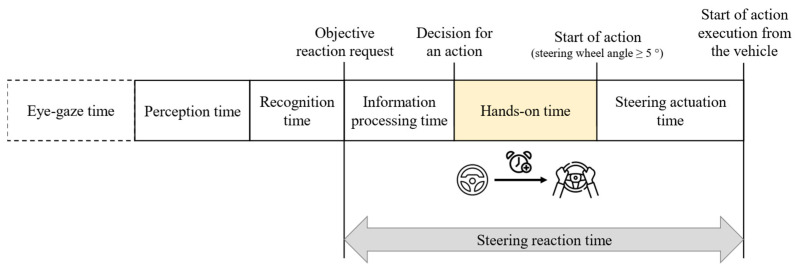
Steering reaction time with additional hands-on time.

Based on this, RQ2 was investigated. In the first step, the type of action performed by the subjects was evaluated. The associated oversteering and, thus, deactivation of the partially automated driving function was defined as a criterion for an accelerating, braking, or steering action performed by the subject. Based on this, the steering wheel angle and the associated steering actuation time were also evaluated. The time the action began was also determined using the variables recorded in Table 1 during a study run. From this, the time required by the subjects from the objective reaction request to the start of the action could be calculated. According to Figure 6, this duration includes an information processing time. This is the time the subject requires from the objective reaction request point to the first recognizable movement of the hands in the videos to apply the required steering wheel angle on the steering wheel. Furthermore, the subjects could take their hands off the steering wheel for up to 15 s. If the subjects still had to move their hands to the steering wheel, the “hands-on time” was also determined from the measured values.

Furthermore, the driving scenario’s criticality was examined to answer RQ3. For a quantitative determination, the distance and time that the subjects had traveled in the oncoming lane until they returned to their lane was examined. In addition, the subjective criticality of the driving scenario was determined for the subjects with the support of the questionnaire. The criticality was divided into five possible answers ranging from “uncritical” to “very critical”.

Furthermore, an analysis of variance (ANOVA) was carried out between the two study groups (experienced ADAS and inexperienced ADAS) for each of the individual steps listed to gain insights into the reaction behavior of the subjects depending on their experience with ADAS.

The second test scenario investigated the driver’s behavior when phantom braking occurred. The time phantom braking occurred was accompanied by a braking deceleration, a visual warning message in the HUD (Figure 5e), and an acoustic warning signal.

This point in time was available for evaluation via a defined trigger in relation to the other measured variables and the synchronized videos and was defined as an objective reaction request point for the subjects. Based on this, the type of action shown by the subjects was evaluated using categorization into “no action (0)”, “accelerator pedal actuation (1)”, “brake pedal actuation (2)”, “accelerator readiness (3)”, and “brake readiness (4)”. In action category 0, the subjects did not perform any foot movements recognizable in the videos before the vehicle came to a standstill (v = 0 km/h). Actions by the subjects in which they put their foot on one of the two pedals but did not press it before the vehicle came to a standstill were assigned to categories 3 and 4. If the vehicle was still braking and pressing the accelerator or brake pedal was recorded, this was assigned to category 1 or 2 accordingly. It was also investigated whether there was a dependency between the type of action performed and experience with ADAS.

**Figure 7:**
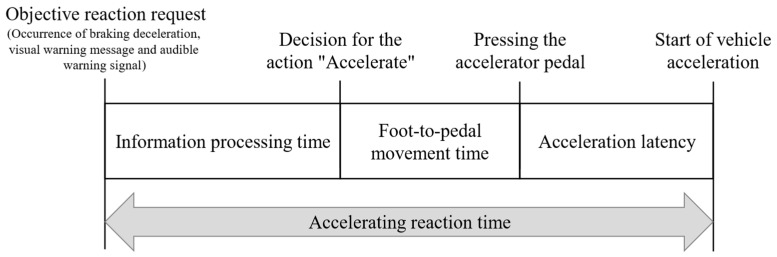
Sequences of an accelerating reaction time from objective reaction request for the driver until the start of vehicle acceleration.

As phantom braking can only be oversteered by pressing the accelerator pedal, the information processing time and foot-to-pedal movement time were determined in the second step according to Figure 7 as part of the specially defined “Accelerating reaction time”. The acceleration latency is the technical time the vehicle requires until it accelerates based on the driver’s request to accelerate. 

The duration from the objective reaction request point to the first recognizable movement of the right foot was measured by video analysis to determine the information processing time. With the beginning of the movement of the right foot, the foot-to-pedal movement time required to press the accelerator pedal was evaluated in the second step.

## 3. Results and Discussion

The following evaluation of the study results and their discussion is conducted separately for test scenario 1 and test scenario 2.

### 3.1. Test Scenario 1: Malfunctioning Behavior of the Activated Partially Automated Driving Function during Cornering

In the first step, it was investigated at what point in time the subjects recognize the occurrence of the system limitation and the associated (imminent) departure from their lane to the oncoming lane when using the partially automated driving function during cornering. To answer RQ1, the initial step was to investigate which subjects had activated the partially automated driving function during cornering. For this purpose, video analysis and measurements were used to filter subjects who consistently oversteered the partially automated driving function and thus deactivated the system. As a result, it was found that only one subject consistently oversteered the system and drove through the curve manually. In the questionnaire, this subject generally had low confidence in such system functions. Consequently, the partially automated driving function was activated in the relevant section of the route for 31 out of 32 subjects, meaning that the data required for the following evaluations was available from 31 subjects. The fact that only one of the 32 subjects oversteered and thus deactivated the system before reaching the test scenario can be explained by the findings of H. Kim et al. [53], N. Strand et al. [15], and M. Dikmen et al. [32]. They found that drivers quickly build trust in the system and underestimate its performance.

Based on the database of 31 subjects, it was investigated whether the subjects recognized the system limitation when the vehicle started crossing the center line (see Figure 3) or when the vehicle had already left its lane into the oncoming lane, and the warning message appeared. For this purpose, the subjects’ answers to the question “How did you recognize that you had to overtake?” in the questionnaire were used. The evaluation of the subjective reaction request showed that all subjects felt they had to oversteer the partially automated driving function when the vehicle was close to or about to cross the center lane. It can be deduced from the results that all subjects independently experienced a reaction request by comparing the target and the vehicle’s actual course. This also implies that the warning message “Take over steering immediately!” that appeared in the HUD on arithmetic mean 0.35 s after crossing the center line was too late and did not serve as a reaction request. There was also no significant difference between the two groups, “Experienced with ADAS” and “Inexperienced with ADAS”. It can be stated that the moment at which the vehicle begins to cross the center line with the left front tire can be used as an objective reaction request point for the driver when reconstructing such accident scenarios if a partially automated driving function has been activated and regardless of the driver’s experience in using the system.

**Figure 8:**
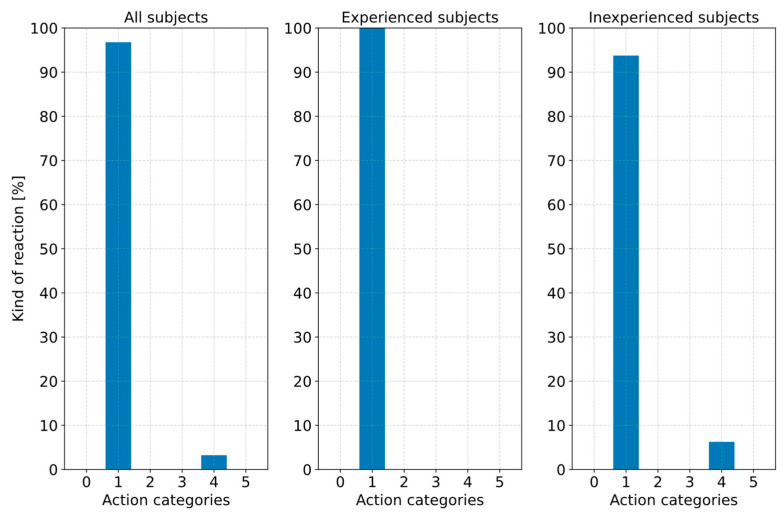
Type of action of the subjects in scenario 1 according to the action categories “no action (0)”, “steering (1)”, “braking (2)”, “accelerating (3)”, “steering and braking (4)” and “steering and accelerating (5)”.

Based on these results, RQ2 was investigated. For this purpose, the first step was to evaluate the type of action performed by the subjects. The video analysis showed that all 31 remaining subjects with the partially automated driving function activated had left their lane into the oncoming lane. However, all subjects showed high attention to the traffic situation due to the recurring request to touch the steering wheel. This behavior can be attributed to the fact that the subjects were not requested to react until they began to cross the center line. When evaluating the reaction behavior in response to deviating from the own lane to the oncoming lane, a distinction was made between the action categories “no action (0)”, “steering (1)”, “braking (2)”, “accelerating (3)”, “steering and braking (4)” and “steering and accelerating (5)”. According to Figure 8, 30 of the 31 subjects (96.7%) showed a pure steering action (1) in response to deviating from their lane to the oncoming lane. A single subject performed a combined steering and braking action (4). It was expected that more subjects would perform a combined steering and braking action. However, the fact that 30 subjects performed a pure steering action can be attributed to an intuitive action to return to their lane as quickly as possible due to the oncoming vehicle. Consequently, it can be assumed that the steering action performed was a trained automated spontaneous reaction independent of ADAS experience. Consequently, the study results do not show that drivers who are inexperienced in using ADAS act differently when deviating from their lane than drivers with ADAS experience. The maximum steering wheel angle executed during the steering maneuver was measured at an arithmetic mean of 89.5° (σ = 36.9°). The median was 91.7° (MAD = 29.3°). The steering actuation time until the maximum steering wheel angle was reached was also determined. The arithmetic mean was 0.65 s (σ = 0.19 s). The median was calculated at 0.64 s (MAD = 0.15 s). This results in an interval for the steering actuation time of 0.59 s to 0.71 s for a confidence level of 90%. Thus, lower steering wheel angular velocities could be found compared to the upper limit in [8] with 400°/s.

**Figure 9:**
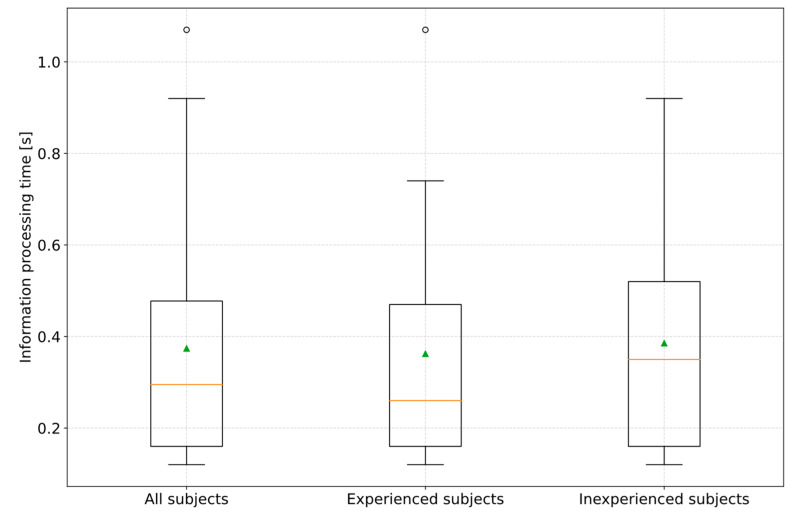
Information processing time in scenario 1 illustrated as boxplot diagram over all subjects, experienced subjects, and inexperienced subjects. The orange line shows the median, and the green triangle shows the mean value.

Based on the findings, the information processing time for the 31 subjects was analyzed. According to Figure 6, the information processing time is the time between the objective reaction request point (crossing the center line) and the driver’s decision to perform a steering action. The decision point was defined by the first recognizable hand movement in the videos. According to Figure 9, the measured values and video analysis showed an arithmetic mean information processing time of 0.37 s (σ = 0.26 s) for all subjects. The median was 0.3 s (MAD = 0.20 s). This results in a confidence interval for the information processing time of 0.25 s to 0.49 s for a confidence level of 90%. The determined time range is comparable to the Cologne reaction time model [18]. Because the subjects were exposed to a high degree of urgency and the situation occurred unexpectedly, the information processing time range can plausibly be used in forensic expert assessments, as these boundary conditions would also be expected in an imminent accident event in real road traffic. Furthermore, a significance level α of 0.05 resulted in a *p*-value of 0.82. This means there is no statistically significant difference in the information processing time of the subjects concerning their experience and knowledge of partially automated driving functions.

Since drivers have the technical ability to take their hands off the steering wheel for up to 15 s when using partially automated driving functions, it was found that 29 out of 31 subjects did not hold their hands on the steering wheel at the objective reaction request point, but on or between their knees. The fact that the 29 subjects did not hold their hands on the steering wheel at the time of the objective reaction request point can be attributed to the fact that all subjects had built up increasing trust in the system over the total distance of 8718 m covered in advance, as the partially automated driving function did not show a single misbehavior during this time. This behavior is consistent with the results of several studies [23,28,29,30], which found that drivers increasingly use the technical option of taking their hands off the steering wheel for up to 15 s the more familiar they become with the partially automated driving function.

**Figure 10:**
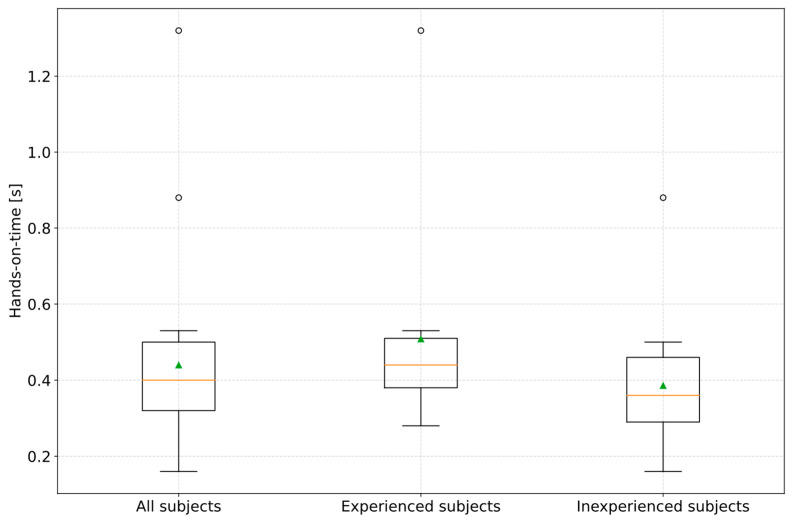
Hands-on time in scenario 1 illustrated as boxplot diagram over all subjects, experienced subjects, and inexperienced subjects. The orange line shows the median, and the green triangle shows the mean value.

Accordingly, a hands-on-time was determined, which follows the information processing time according to Figure 6. This is the time drivers require from the first movement of the hand to the start of the steering action. For the remaining 2 of 31 subjects, it was found that although they did not touch the steering wheel directly and oversteered the system, they kept their hands open on the steering wheel, which means that in these cases, the measurement of a hands-on time was not practical. Figure 10 illustrates that all subjects showed an arithmetic mean hands-on time of 0.44 s (σ = 0.23 s). The median was 0.4 s (MAD = 0.14 s). This results in a confidence interval for the hands-on time of 0.34 s to 0.54 s for a confidence level of 90%. The results also showed that there is no need to differentiate between the level of experience of subjects with ADAS. ANOVA revealed a *p*-value of 0.20 at a significance level α of 0.05 between the experienced and inexperienced subjects. Due to the oncoming vehicle, there was a high urgency to act, so the subjects showed the fastest possible reaction behavior. Following the explanations of existing studies, D. Damböck found a hands-on time of 0.3 s. However, where the subjects’ hands were located at the start of the first movement was not specified. As the 0.3 s is approximately at the lower limit of the confidence interval determined, it is possible that the subjects in D. Damböck’s study could have held their hands below the steering wheel, which would have resulted in shorter hands-on durations being measured. D. Damböck also did not clearly define his hands-on time. It seems to be possible that the measured duration ended with the first contact of a hand on the steering wheel. In this study, the start of steering corresponded to a measured steering wheel angle of 5°. This is the steering wheel angle required for a conscious system oversteering by the subject.

**Figure 11:**
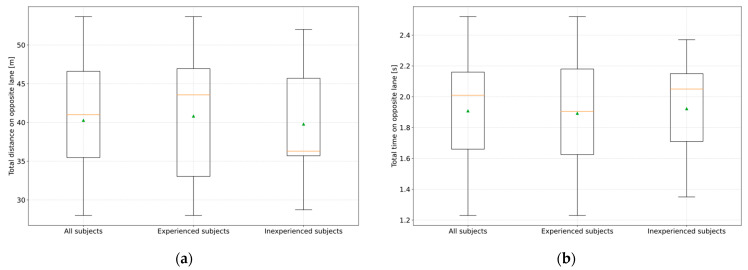
(**a**) Total distance in the opposite lane; (**b**) total time in the opposite lane. The orange line shows the median, and the green triangle shows the mean value.

The criticality of the scenario was also examined to answer RQ3. Overall, the results show that it is unavoidable for the driver to leave their lane during cornering when a corresponding limit of the partially automated driving function is reached without being warned in advance by the system. In addition, it was found that leaving their lane can end in an overall very critical and, therefore, accident-prone driving situation, as the subjects covered an arithmetic mean distance of 40.3 m (σ = 8.11 m) in the oncoming lane until the vehicle was back in the driver’s own lane according to Figure 11a. The arithmetic mean was 40.8 m (σ = 9.44 m) for the inexperienced ADAS subjects and 39.8 m (σ = 7.28 m) for the experienced subjects. Furthermore, it can be seen from the diagram that the distance traveled in the oncoming lane by the experienced subjects is almost identical to that of the inexperienced subjects. This is also confirmed by analyzing the study results using an ANOVA (Python 3.10.9 with the module Scipy 1.10.0). At a significance level α of 0.05, a *p*-value of 0.80 is obtained between the experienced and inexperienced subject groups.

The driving time in the oncoming lane was measured for all subjects in the arithmetic mean of 1.91 s (σ = 0.37 s), as shown in Figure 11b. Again, no significant difference between the two groups could be detected (*p*-value = 0.88). Furthermore, the subjects were faced with a high urgency to return to their lane due to the oncoming vehicle, which was already visible at the beginning of the curve entry and before crossing the center line. Consequently, it can be stated that the measured values represent the fastest possible return times for drivers. It can be concluded that it is not experience with ADAS that is decisive for the distance traveled or the time spent in the oncoming lane, but rather the driving speed when crossing the center line. Furthermore, it can be deduced from the results for the reconstruction of oncoming lane accidents that in the event of a collision area between the apex of the curve and up to 40.8 m afterward in the oncoming lane, it must be considered that the deviation of the vehicle from the driver’s own lane and consequently the oncoming lane accident may have been caused by a corresponding misbehavior of the partially automated driving function.

**Figure 12:**
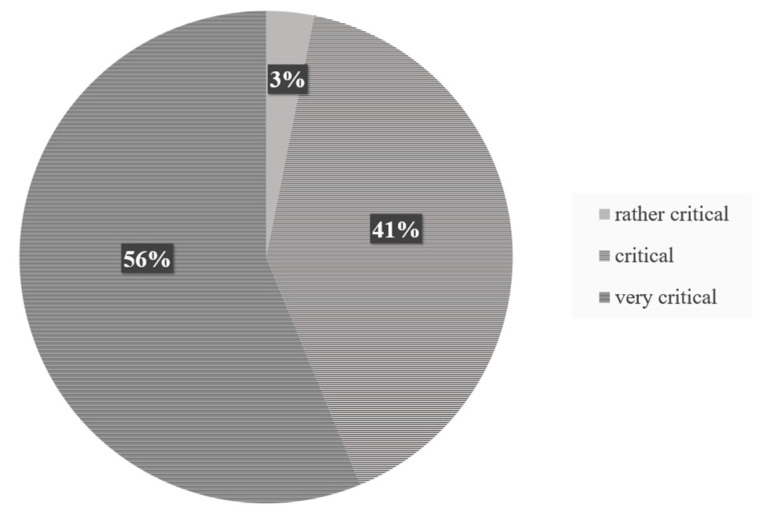
Subjective criticality of the subjects. Ratings have been very critical, critical, rather critical, uncritical, and very uncritical.

The pie chart in Figure 12 illustrates that the driving scenario was critical for the subjects in general. 56% of the subjects stated in the questionnaire, on the question “How critical was the situation for you?” that it was very critical. A further 41% of the subjects rated the situation as critical. Only 3% of the subjects considered the situation to be rather critical. None of the subjects selected the “uncritical” or “very uncritical” answer field. This classification of the criticality of the driving scenario can be attributed, on the one hand, to the fact that the subjects were driving in the oncoming lane for an arithmetic mean of 1.91 s when a vehicle was approaching. Furthermore, leaving their lane was unavoidable, as there was no warning message from the system to take over control of the vehicle before reaching the curve apex, which could have led to an earlier reaction request and thus helped to mitigate the driving scenario. The need for earlier system warnings was also noted by Kim et al. [53] to avoid such accident-prone situations.

### 3.2. Test Scenario 2: Phantom Braking

In test scenario 2, the reaction behavior of drivers in the event of system-initiated phantom braking when using a partially automated driving function was investigated. In the first step, the objective reaction request point was examined. For this purpose, the hypothesis was formulated that the subjects received a reaction request when the visual warning appeared on the HUD, when the acoustic warning message was emitted, or when the brakes were applied. The questionnaire’s evaluation of the question “How did you recognize that you had to intervene?” showed that 26 out of 32 subjects experienced an objective reaction request with a visual or acoustic warning message. Four other subjects stated that they only recognized that they had to act when they felt the start of braking. The remaining two subjects stated that they did not even recognize that they had to intervene. The evaluations thus show that a visual or acoustic warning message, usually issued simultaneously during an AEBS-initiated braking process, whether faulty or not, can plausibly be used as an objective reaction request point in the avoidability analysis.

**Figure 13:**
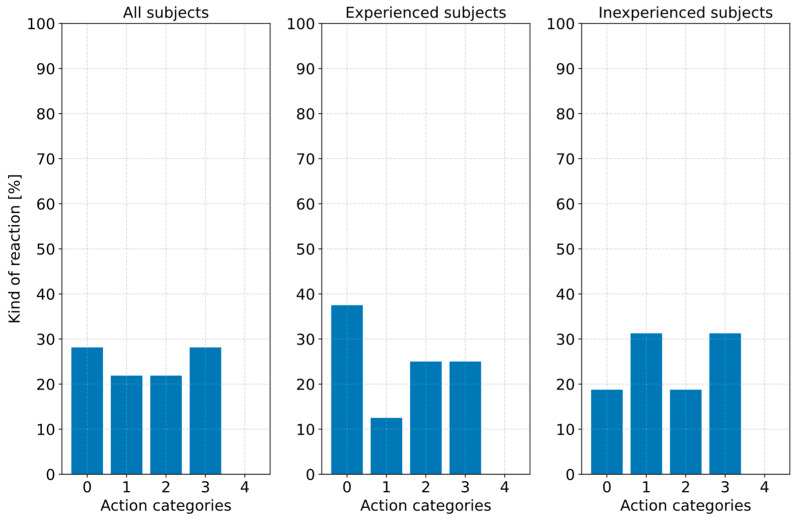
Distribution of the kind of action for the subjects in reaction to phantom braking according to the action categories “no action (0)”, “pressing the accelerator pedal (1)”, “pressing the brake pedal (2)”, “willingness to accelerate (3)”, “willingness to brake (4)”.

The kind of action performed by the subjects was evaluated as a function of their experience with ADAS. Figure 13 shows that 28.1% (*n* = 9) of the subjects showed no movement of the right foot and thus no action (action category 0). This value comprises 37.5% (*n* = 6) of the 16 experienced subjects with ADAS and 18.75% (*n* = 3) of the 16 inexperienced subjects. Another 21.9% (*n* = 7) of the subjects performed a braking action (action category 2) in response to the phantom braking. This included four subjects with experience with ADAS and three with no experience with ADAS. The braking reaction behavior could be attributed to a trained automated reaction pattern without analyzing the vehicle environment in detail and critically questioning the executed system behavior. In addition to the nine subjects who did not perform any action, nine others showed an action until the vehicle reached a standstill but only a willingness to accelerate (action category 3). This means the subjects put their right foot on the accelerator pedal but did not press it. None of the subjects were willing to brake (action category 4). The fact that only 21.9% (*n* = 7) of the subjects ultimately oversteered the phantom braking by pressing the accelerator pedal (action category 1) is particularly revealing. Furthermore, it can be seen from Figure 13 that there is no significant difference between the groups, although the experienced subjects had also read an information sheet based on the vehicle manufacturer’s manuals before starting the study, which described how such system behavior could be oversteered. The conclusion could be drawn that drivers in such a surprising and possibly challenging situation cannot remember the information they have read and act purely intuitively in the short sequence of events, which did not include pressing the accelerator pedal in a driving situation that seemed critical to them. This uncertainty could also explain why nine subjects put their foot on the gas pedal but ultimately did not press it.

The evaluations of the kind of action showed that only 7 out of 32 subjects were able to oversteer the phantom braking by pressing the accelerator pedal before the vehicle came to a standstill (v = 0 km/h) to avoid a rear-end collision with the following truck. Consequently, the data were only available from these subjects in sufficient form to determine an information processing and foot-to-pedal movement time until the accelerator pedal was pressed as part of the accelerating reaction time based on the objective reaction request point. The evaluations of the information processing time showed that the seven subjects required an arithmetic mean of 0.82 s (σ = 0.22 s) from the objective reaction request point to the first movement of the right foot. The median was calculated at 0.96 s (MAD = 0.19 s). This results in a confidence interval of 0.57 s to 1.07 s for a confidence level of 90% for the information processing time as a sequence of the accelerating reaction time. The variance analysis revealed no significant difference between the two groups at a significance level α of 0.05 (*p*-value = 0.50). The measured information processing time range of 0.32 s to 0.58 s is more than twice as high as the range in the first test scenario. This could be attributed to the possibility that the drivers first search for the warning reason in the vehicle environment and conduct a target/actual comparison until they decided to act. However, this hypothesis must be verified using further studies with a larger sample size.

All subjects showed a foot-to-pedal movement time of 1.09 s (σ = 0.69 s) on the arithmetic mean. The median was 1.04 s (MAD = 0.52 s). This results in a confidence interval of 0.38 s to 1.8 s for a confidence level of 90% for the foot-to-pedal movement time as a sequence of the accelerating reaction time. ANOVA showed no significant difference between the two groups at a significance level α of 0.05 (*p*-value = 0.44). The results show that the confidence interval of the foot-to-pedal movement time is significantly higher than the classic one, with 0.15 s to 0.3 s. The deviations can be explained in particular by the fact that five out of seven subjects hesitated to press the accelerator pedal or, in extreme cases (2.08 s), even switched back and forth between the gas pedal and brake pedals until the accelerator pedal was finally pressed.

Since the results on information processing and foot-to-pedal movement time are only based on a database of seven subjects, the findings cannot be used as scientifically substantiated fundamental data for forensic accident analysis. Nevertheless, the investigations were able to ascertain that a driver’s pressing of the accelerator pedal cannot coincide with the objective reaction request point and that a corresponding accelerating reaction time must be applied in the avoidability analysis. Furthermore, it must be remembered that an acceleration reaction is not a classic steering or braking reaction that has been practiced over many years in daily road traffic, resulting in developed automatisms. The study results indicate that significantly longer accelerating reaction times compared to steering or braking reaction times should be applied to drivers.

## 4. Conclusions

In this work, a simulator study was conducted with 32 subjects on driver reaction behavior in the event of a malfunctioning behavior of partially automated driving functions. The aim is to generate fundamental data for forensic accident reconstruction usable in expert reports in court in the future.

In the first of the two test scenarios, the reaction behavior of the drivers in the event of a system limitation of an activated partially automated driving function during cornering was investigated. The reason for the limitation was reaching the curve limit speed limited by the specified maximum lateral acceleration of 3 m/s^2^ in the area of the curve apex. The driver was not requested to take over vehicle control in advance either. In summary, it was found that all subjects (*n* = 31) in which the partially automated driving function was activated during cornering were unable to avoid deviating from their lane into the oncoming lane. Furthermore, the study results showed that the subjects received an objective reaction request by comparing the vehicle’s target and actual course when the vehicle began crossing the center line. As a result, the contact of the left front tire with the center line can be plausibly defined as an objective reaction request point for the driver in the avoidability analysis. Consequently, using the warning message as an objective reaction request point for the driver would only be conceivable if this occurred before the center line was crossed. Of the subjects, 96.7% (*n* = 30) showed a pure steering action as a reaction to deviating from their lane to the oncoming lane. A range of 0.59 s to 0.71 s could be calculated for a confidence level of 90% for the steering actuation time required to return to the driver’s own lane. For the information processing time from the objective reaction request point to the start of a hand movement, a time range of 0.25 s to 0.49 s could be found for a confidence level of 90%. For hands-on time, the interval was 0.34 s to 0.54 s. This results in a total steering reaction time range of 1.18 s to 1.74 s. Consequently, it can be concluded that the previously used steering reaction time range of 0.6 s to 1.2 s must be increased by the time component of the hands-on time range. Compared to the results of [22], it could not be determined that drivers who know the possible system limits and have experience with such a system would also have a reaction time similar to that of manual driving. This is also comparable with the findings of Shen [12,13] and Powelleit [20], which also determined longer driver reaction times when a partially automated driving function is activated. In comparison to manual driving, the reason for longer reaction times can also be attributed to the fact that all subjects deviating from their lane used the technical option of taking their hands off the steering wheel. This “out-of-the-loop” behavior is comparable to the findings of the studies [23,24,25,26] described in the related work. Accordingly, it can be concluded that the subjects had built up a high level of trust in the system and were surprised by the event at the same time. To summarize, it can be concluded for the forensic accident analysis that a change in the driver’s reaction behavior compared to manual driving must be considered in the case of an activated partially automated driving function during an accident.

In the future, it will be possible to determine whether a partially automated driving function has been activated using the ADAS activities stored in the EDR [16]. Furthermore, it can be deduced from the test results that the measured steering reaction time range would have been 0.84 s to 1.2 s if no hands-on time range were applied. Consequently, consideration should be given to generally increasing the lower limit of the driver reaction time by at least 0.24 s to 0.84 s if a partially automated driving function was activated, even if the driver kept his hands on the steering wheel. This may be related to a longer driver information processing time, which is in alignment with the findings in [21]. In addition, it was found that the subjects, despite a high degree of urgency, remained in the oncoming lane for an arithmetic mean of 1.91 s (σ = 0.37 s) after crossing the center line until they returned to their lane, which in reality can lead to severe accidents. These values need to be supported with real vehicle tests, since the steering behavior of a simulator does not fully represent real vehicle behavior.

Also, the study results from the first scenario revealed that the human driver is generally unsuitable as a monitoring instance for the functionality of a partially automated driving function if the driver has to recognize the occurrence of a hazardous situation via the target/actual comparison by himself. This can be reconciled with the results of [15,33]. Consequently, the behavior of partially automated driving functions should be improved to the extent that they inform the driver about the possibility of a malfunctioning behavior occurring before entering the cornering area, but at the latest at the time of the determined upper limit of the steering reaction time (1.74 s). From a technical point of view, visual or acoustic warning messages can be considered a reaction request point in forensic accident analysis [54], even if drivers often ignore them [55]. Furthermore, V2X technology could also reduce criticality and thus increase road safety [56]. Other road users or the infrastructure could warn the driver before reaching a tight curve that the system limits could be reached and that a take over control of the vehicle should be performed as a precaution. Furthermore, the survey of accident analysts showed that 86% (*n* = 89) of the participants think that an additional extended driver reaction time can be expected if a warning message is used as an objective reaction request point. Indeed, the driver must first understand the warning message as part of the information processing time [10]. There is also the possibility that the driver will first search for the warning reason in the vehicle environment and conduct a plausibility check of the warning with reality via the target/actual comparison before following the message and ultimately deciding to act. Accordingly, there is a need for further research on whether drivers will show an earlier start of action in the case of a warning message before cornering.

The second test scenario examined the reaction behavior of the subjects in the event of phantom braking initiated by the AEBS and whether they were able to oversteer the system’s misbehavior by pressing the accelerator pedal before the vehicle came to a standstill while a partially automated driving function was activated at the same time. The results show that 26 out of 32 subjects experienced an objective reaction request with the visual or acoustic warning message that accompanied the start of braking. As a result, the objective reaction request point for the driver can be plausibly and comprehensibly applied in the avoidability analysis when the visual or acoustic warning message occurs. Only 7 out of 32 subjects could oversteer the braking action (phantom braking) incorrectly executed by the AEBS by pressing the accelerator pedal before the vehicle came to a standstill while a partially automated driving function was activated. For the seven subjects, a confidence interval of 0.57 s to 1.07 s was found for the information processing time as a sequence of accelerating reaction time at a confidence level of 90%. A confidence interval of 0.38 s to 1.8 s could be calculated for the foot-to-pedal movement time as a sequence of the accelerating reaction time. The tolerances can be explained by the fact that five out of seven subjects hesitated to press the accelerator pedal or, in extreme cases (2.08 s), even switched back and forth between the gas pedal and brake pedal with their right foot until the accelerator pedal was finally pressed. Of the seven subjects who oversteered the phantom braking by pressing the accelerator pedal, five subjects had no experience with ADAS, and two subjects had experience with ADAS. Furthermore, the subjects with ADAS experience had to read an information sheet describing the possibilities of oversteering a faulty system behavior before starting the study. However, at a significance level α of 0.05, the study results showed no significant difference in reaction behavior between the subjects with ADAS experience and those who were inexperienced with ADAS and had not read the information sheet. This, in turn, implies that drivers in such a critical driving situation do not remember what they have read but rather react reflexively. Consequently, such situations should be trained in practice to develop reflex-like automatisms for pressing the accelerator pedal, similar to a classic reaction pattern (braking/steering). In the questionnaire, 27 out of 32 subjects affirmed that the correct reaction behavior in the event of a phantom braking maneuver should not be trained solely in real road traffic but rather during driving school training. Kim. et al. [53] also came to the conclusion that the handling of the occurrence of critical driving situations by Level 2 systems must be trained. The authors conducted a study in which naturalistic driving data was collected from 50 participants who drove private vehicles with partially automated driving systems for 12 months. The study captured a number of scenarios in which ADAS failed to meet driver expectations in typical driving situations, such as cornering.

Contrary to expectations that even the most informed drivers could not oversteer the system’s misbehavior, it must be pointed out that the results for accelerating reaction times are only based on a database of seven subjects, meaning they cannot be used as scientifically substantiated fundamental data for forensic accident analysis. Consequently, the accelerating reaction time drivers require must be investigated in further studies.

The results from the two test scenarios generally indicate that driver training in ADAS usage through manuals is inadequate. To improve this, manufacturers could, for example, show an explanatory video in the infotainment system before activating an ADAS for the first time. This could also be repeated at specific time intervals. Whether an explanatory video has an advantage over manuals regarding how ADAS is used must be investigated in further studies. However, an explanatory video could ensure that the driver at least receives instructions on how to use the ADAS if only a successful viewing of the video leads to activation of the ADAS.

The study results also indicate that the driver cannot fulfill his according to [9] presumed responsibility to immediately take control of the vehicle in the event of a system malfunction since an adequate reaction time has to be considered. It was also found that a longer reaction time must be applied compared to manual vehicle control, even if the drivers had read the manual and, thus, been informed about the possible system limits and their overriding possibilities. In addition, the interactions between the driver and the ADAS should be set into a fair context concerning the real possible reaction behavior of the driver while considering the “out-of-the-loop” problem due to trust in the system’s performance. Therefore, the currently prevailing legal view of the expected reaction behavior of drivers for the avoidability assessment of an accident should be reconsidered as soon as faulty ADAS behavior is responsible for the occurrence of a hazardous situation and as long as drivers are not sufficiently trained in dealing with such situations, especially in overruling phantom braking by pressing the accelerator pedal. However, as the study is based on a sample size of 32, further (field) studies must be conducted to support the results. As data on the ADAS activities that took place during an accident will be stored in the EDR due to Revision 1, it can be assumed that ADAS misconduct will increasingly play a central role in court proceedings. This results in an urgent need for further research as well as action within legislation and jurisdiction.

## Figures and Tables

**Table 1:** Recorded variables during a study run.

Variables	Recording
Accelerator pedal position	CarMaker
Brake pedal position	CarMaker
Steering wheel angle	CarMaker
Vehicle velocity	CarMaker
Vehicle longitudinal acceleration	CarMaker
Vehicle lateral acceleration	CarMaker
Vehicle trajectory on the road	CarMaker and Cameras
Distance traveled	CarMaker
Travel time	CarMaker
System information	CarMaker and Cameras
Driver movements	Cameras
Distances to other road users	CarMaker
Subjective study experiences	Questionnaire

## Data Availability

The data presented in this study are available on request from the corresponding author. The data are not publicly available due to privacy restrictions.
